# Ebola virus disease and the veterinary perspective

**DOI:** 10.1186/s12941-015-0089-x

**Published:** 2015-05-28

**Authors:** Semra Gumusova, Mustafa Sunbul, Hakan Leblebicioglu

**Affiliations:** Department of Virology, School of Veterinary Medicine, Ondokuz Mayis University, Samsun, Turkey; Department of Infectious Diseases and Clinical Microbiology, Medical School, Ondokuz Mayis University, Samsun, Turkey

**Keywords:** Ebolavirus, Ebolavirus disease, Prevention, Animals, Human and veterinary perspective

## Abstract

Ebola virus disease (EVD) is a potentially fatal haemorrhagic disease of humans. The last and most serious outbreak of Ebola virus (EBOV) started in December 2013 in West Africa and also affected other continents. Animals such as fruit bats and non-human primates are potential sources of EBOV. This review highlights the clinical features of EVD in humans and animals and addresses the public health implications of EVD outbreaks from the veterinary perspective.

## Introduction

Ebolavirus disease (EVD) was first described in the Democratic Congo Republic (DRC) in 1976. 318 cases were reported in that outbreak due to close contact and use of contaminated needles at the hospital, 280 of whom (88 %) died [1]. Cases and outbreaks were reported in the ensuing years from a number of different countries in central Africa [2]. The latest and most serious outbreak started in December 2013 in Guinea and affected other African countries such as Sierra Leone, Liberia, Mali, Nigeria and Senegal, with cases exported to other countries in Europe and North America. The total number of cases had reached 26,593 as of May 11, 2015, 11,005 of them had died [3].

The case fatality rate has ranged from 60–70 % in the three most affected countries (Sierra Leone, Liberia and Guinea) [4]. Nosocomial infections are common due to person-to person transmission in the health care setting both to staff and other patients. The disease has been contracted by 868 healthcare workers, and of whom 507 have died to date [3]. The World Health Organization (WHO) reported that the localised outbreaks were finished on the 17th of October in Senegal, 19th of October in Nigeria, and on 2nd of December in Spain. Liberia has now just been declared EVD free, whilst limited community transmission continues in Sierra Leone and Guinea [3].

The disease is transmitted to humans by direct contact with infected animals or body fluids. The chain of transmission continues with direct contact with infected persons or their blood or body fluids [5]. Transmission of the Ebola virus by airborne particles has been hypothesized in animal models but not demonstrated in humans [6]. Healthcare workers in direct contact with infected persons or their body fluids without personal protective equipment are at particular risk. People caring for patients at the hospital or at home, and those that have had direct contact with those who died, often due to traditional funeral practices are also at high risk [7]. A number of studies have shown the absence of transmission of EVD to persons living in the same household with the patient, but who have not had direct contact [8].

EVD is quickly progressive disease with multisystem involvement, causing bleeding and coagulopathy. The most frequent clinical findings are fever, nausea, vomiting, diarrhea and fatigue [9]. After an incubation period five to nine days with a range of 1–21 days, there is generally an acute presentation with flu-like symptoms such as fever, chills, myalgia and headache at the early phases of the disease, after which gastrointestinal symptoms such as abdominal pain, nausea, vomiting and diarrhea are seen. [4, 10] Cough and sore throat may be present and a maculopapular rash may occur 5–7 days after the onset of symptoms, but is infrequently seen in darker pigmented skin. Bleeding as an oozing from venepunctures or cannula sites along with petechiae and ecchymosis and mucous membrane bleeding occur [4]. The causes of death are multifactorial and include massive haemorrhage (bleeding occurs in up to 50 %), hypovolemia and electrolyte imbalance, severe sepsis and multi-organ failure [11]. Survivors often show improvement on the 6th–11th days, co-inciding with the development of neutralizing antibodies [12].

Ebolavirus (EBOV) is generally detected in blood when fever and symptoms first occur. The preferred diagnostic test is RT-PCR in blood, due to the presence of high viremia at the acute phase. Viral RNA reaches its peak at or around the 5th day after the onset of symptoms and is generally positive in blood for as long as febrile clinical disease continues. PCR study may be done in serum, plasma, whole blood or body fluids [13].

IgM type antibodies may be detected in blood samples in the days following onset of disease symptoms with antigen-capture enzyme-linked immunosorbent assay (ELISA), but its sensitivity is lower than RT-PCR. Virus blood cultures are generally positive in the early phases with Vero E6, but this should not be done without a level-4 bio-safety laboratory [13].

Treatment is essentially supportive including aggressive volume and electrolyte replacement, oral and intravenous nutrition and symptomatic treatment is important. There is no approved anti-viral treatment for EVD. [14] Several agents currently undergoing clinical trials have anti-viral activity against EBOV. Drugs such as clomiphene, toremifene, favipiravir, brincidofovir and imatinib have been tried in EVD. Favipiravir, which is a derivative of pyrazine carboxamide was found to be effective against EBOV in animal models. Studies on the protective vaccine are continuing, with promising preliminary findings [15]. Immune based therapy under investigation as treatment options including convalescent plasma and monoclonal antibodies [16].

## Animals and ebolavirus

The genus *Ebolavirus* is divided into five distinct species, *Zaire ebolavirus* (Ebola virus, EBOV), *Sudan ebolavirus* (Sudan virus, SUDV), *Tai Forest ebolavirus* (Tai Forest virus, TAFV), *Bundibugyo ebolavirus* (Bundibugyo virus, BDBV), and *Reston ebolavirus* (Reston virus, RESTV [17]. Virus can infect non-human primates (NHPs) (chimpanzee, monkey, macaque, orangutan and baboon) [18, 19], fruit bats [20], pigs [21], dogs [22], rodents [23], porcupines [24] and a range of laboratory animals (guinea pigs, mice, and hamsters) [25]. Studies on arthropods have revealed that these are not vectors of EBOV [26].

## Epidemiology

The important filovirus source for humans are animal carcasses in the forest [27]. According to Changula et al., [28] the cause of the increasing and frequent outbreaks was due to human eradication of forests. The increasing contact of the human and virus reservoir, the combined with its virulence has enhanced EVD epidemics [29]. The relationship of climate change and infectious diseases may also affect wildlife habitats and increase the frequency of disease outbreaks [30].

The onset of EVD usually occurs at the beginning of the dry season. It has been suggested that dry season has been extended due to deforestation of the area over recent decades, and environmental reports have suggested an extremely dry season in Guinea during the period of the epidemic. Although uncertain, researchers have suggested that dry climatic conditions increase the number or availability of EBOV–infected bats and/or the frequency of human contact with them [31, 32].

## Role of animals in virus transmission

EBOV has infected a number of animal species by natural or experimental methods, but exactly which animal species plays a role in the transmission of infection to humans is still being investigated [33]. During human outbreaks, research has determined that EBOV is zoonotic and bats have served as reservoirs and the source for infection [27]. Bats do not show clinical infection when infected with EBOV [34]. REBOV has been detected in pigs and antibodies demonstrated in pig farmers in the Philippines. EBOV replication in pigs and zoonotic transmission from pigs to human has been described [20].

The Food and Agricultural Organization of the United Nations has warned the public about EVD outbreaks being related to consumption of bat meat. Ebola virus disease outbreaks in humans have also been associated with infected apes, which are hunted for food [32]. Analysis has shown that gorilla, chimpanzee and duiker carcasses could be the main sources of human infections [35]. The potential epidemiological role of cats and dogs is not known, and so far studies have revealed that dogs may become infected with EBOV, but do not demonstrate clinical symptoms [36].

## Clinical symptoms of EVD in animals

Only a few of the 23 epidemics in the world have had destructive effects on NHPs [37], considered to be a reservoir for the virus in the wild [38]. The incubation period of their infection is 2–14 days [39], and clinical signs are anorexia, cough, weakness and bleeding, with death (mortality usually 100 %) occuringin a few days [40]. Fruit bats can be infected with EBOV but do not get clinical disease, and are deemed a main reservoir species for EBOV [27]. It’s known that pigs are the only species of livestock to be susceptible to EBOV, but have not been shown to be naturally infected with EBOV in the current outbreak in West Africa. However, pigs have been infected with EBOV (Zaire ebolavirus - the species in the current West African outbreak), and infection has been transmitted from pigs to monkeys, experimentally. The clinical symptoms were not observed during experimental infection in pigs [21]. The natural Reston ebola virus infection (RESTV) of pigs have been found in the Philippines and China, but RESTV does not cause human illness. All of the RESTV infected pigs were also infected with the porcine reproductive and respiratory syndrome virus (PRRSV), so it was not clear whether RESTV was responsible for any clinical signs or not [20]. EVD clinical signs have not been observed in cats and dogs.

## Diagnosis

Antigen-capture enzyme-linked immunosorbent assay (ELISA), IgM ELISA, IgG ELISA DNA amplification, sequencing and virus isolation are used for the diagnosis of EVD in animals [32].

## EVD and animal models

Animal models are important for understanding EVD pathogenesis, licensing new drugs and vaccines and for designing effective control measures for EBOV. NHPs, mouse, guinea pig and Syrian golden hamster models have been used for evaluating interventions against EVD. NHPs models exhibits similar symptoms, including hemorrhage and shock in EVD patients, and these are the gold-standard for the study of EVD pathogenesis. However, cost, ethical and handling problems limits their large-scale use at studies. Small animal models are also suitable for pathogenesis research and the intial evaluation of the efficacy of vaccines and therapeutics [25].

Mouse models were adapted with wild-type EBOV by serial passage [41]. EBV antigen was detected in the spleen and hepatocytes but clinical symptoms including fever and cutaneous rash, and laboratory signs such as intravascular coagulation (DIC), prolongation of prothrombin time (PT) and activated partial thromboplastin time (aPTT) were not observed in mouse models [42]. Guinea pig has been infected with ZEBOV by sequential passage and presented EVD-like symptoms such as fever, anorexia, dehydration [43], and demonstrated progressive prolongation of the PT and aPTT [44] but rash does not develop in these animals [42]. The Syrian golden hamster model was infected with mouse-adapted EBOV and presented the most similar symptoms of EVD to humans and NHPs [25]. For this reason, it has been used for the study of EVD interventions [45]. The newly developed hamster model is used to confirm drug’s efficacy in NHPs to control coagulopathy, and this model will probably replace mice and guinea models as an alternative model for pathogenesis studies and efficacy testing [42].

## Prevention

The control of virus transmission, through quarantine (suspected cases) and isolation of infected persons is necessary for EVD prevention. EVD licensed vaccines are still not available, but a number of vaccine approaches have been developed for Ebola virus infection in animal models, and are undergoing clinical trials. Recognising that EBOV is transmitted through wild animals such as bats, monkey and gorillas, it is recommended to avoid direct contact with bat and non-human primate blood, bodily fluids and raw meat and meat should be properly cooked before consumption.

During the ongoing EVD epoidemic a pet dog belonging to a Spanish healthcare worker who was infected with EBOV, was euthanased. According to Spanish researchers, implementation of euthanasia to dogs supsected of infection was a public health obligation to protect the public. Another suspected EBOV dog case was reported in Dallas, Texas. In this case, the infected nurse’s dog was tested and then quarantined. Spanish researchers have argued that the dog in Texas was potentially less contagious than the Spanish dog, but the US government has suggested that effective quarantine is sufficient [46].

Veterinarians should warn pet owners who travel to areas in the epidemic, that EVD is a zoonotic disease and dogs could pass the virus on to humans through biting, licking and direct contact with their urine or feces. Pets exposed to an infected individual’s blood or body fluids have potential risk and it should be reviewed by a veterinarian as soon as possible to implement quarantine. Pet owners infected with EVD should be aware that their animal may become infecetd and although showing no symptoms, may be effective in spreading the disease.

## Conclusion

Most of the current emerging viral diseases including rabies, avian influenza, EVD, and hantavirus are zoonoses. As a result, there is direct interface of human and veterinary medicine in many area. To fully understand the wildlife reservoir of zoonoses, surveillance systems for human and animal infection are erquied and the information from such systems shared. The interaction of human, animal, and environmental health is at the heart of one health [47].

Further research into the epidemiology and pathogenesis of EVD is needed to improve our understanding of the disease, diagnostic methods, vaccines and treatment. When an EVD outbreak has started, with rapid virus transmission and spread research is challenging and public health interventions predominate. Medical and veterinary authorities must research EVD together to understand and decrease the transmission risk from animals to humans and humans to animals. They must determine all the reservoir animals to reduce the rate of transmission, and this interdisciplinary collaboration must continue to allow the rapid diagnosis and control of wildlife reservoirs for effective prevention and support of EVD outbreaks. The gorilla and chimpanzee populations in the world are also dircetly at risk, and this collaboration to protect ape populations should be an important priority for wild-life continuity [37]. Testing and quarantine of suspected animal cases, especially domestic pets, is much more humane nearby with benefits of eliciting the zoonotic and reverse zoonotic aspects of the infection. All countries are at risk for EVD in the globalizing world but early detection and effective medical interventions may control the disease.

## Abbrevations

aPTT: activated partial thromboplastin time
BDBV: Bundibugyo ebolavirus
DIC: Intravascular coagulation
DRC: Democratic Congo Republic
EVD: Ebola virus disease
EBOV: Ebola virüs
ELIZA: Enzyme-linked immunosorbent assay
NHPs: Non-human primates
PT: Prothrombin time
RESTV: Reston ebolavirus
RT-PCR: Reverse-transcriptase polymerase chain reaction
PRRSV: Porcine reproductive and respiratory syndrome virus (PRRSV)
SUDV: Sudan ebolavirus
TAVF: Tai Forest ebolavirus
WHO: World Health Organization
